# HGF/c-MET抑制剂治疗非小细胞肺癌的研究进展

**DOI:** 10.3779/j.issn.1009-3419.2015.04.09

**Published:** 2015-04-20

**Authors:** 涛 蒋, 彩存 周

**Affiliations:** 200433 上海，上海市肺科医院 Shanghai Pulmonary Hospital, Shanghai 200433, China

**Keywords:** HGF, c-MET, 肺肿瘤, 抑制剂, HGF, c-MET, Lung neoplasms, Inhibitor

## Abstract

分子靶向治疗在晚期非小细胞肺癌（non-small cell lung cancer, NSCLC）患者的治疗中起着越来越重要的作用。肝细胞生长因子/c-MET（hepatocyte growth factor/c-MET, HGF/c-MET）信号通路在NSCLC的发生、发展及NSCLC患者对表皮生长因子受体抑制剂（epidermal growth factor receptor tyrosine kinase inhibitor, EGFR-TKI）的耐药方面都起着重要的作用，其已经成为NSCLC分子靶向治疗的又一热点。*c-MET*扩增或过表达被认为可能是继EGFR和间变淋巴瘤激酶（anaplastic lymphoma kinase, *ALK*）融合基因之后，NSCLC又一特征性的基因突变。HGF/c-MET抑制剂也在临床前的研究中取得良好的抗肿瘤效果。近期公布的一些Ⅱ期/Ⅲ期临床研究的数据显示，HGF/c-MET抑制剂可以使部分c-MET扩增或过表达的NSCLC患者生存获益。因此，本文就HGF/c-MET抑制剂治疗NSCLC的研究进展做一综述。

## Abstract (en)

Molecular targeted therapy has become more and more important in the treatment of non-small cell lung cancer (NSCLC). HGF/c-MET plays the pivotal role in the growth, development and tolerance to epidermal growth factor receptor tyrosine kinase inhibitor of NSCLC. Moreover it has become another heat point in the molecular targeted therapy of NSCLC. c-MET amplification or high expression was deemed to another significant gene modification beyond EGFR and ALK. In the preclinical studies, HGF/c-MET inhibitors have showed the promising anti-tumor effect. Recently, some phase Ⅱ/Ⅲ clinical trials have proved that these inhibitors could improve the survival of patients with NSCLC. Hence we performed this review to elaborate the research progress of c-MET inhibitor in the treatment of NSCLC.

## 1 概述

肺癌是全球发病率和死亡率最高的恶性肿瘤之一，其中非小细胞肺癌（non-small cell lung cancer, NSCLC）患者约占总数的85%-90%^[1]^。以铂类为基础的标准化疗可以改善患者的总生存期（overall survival, OS）和无进展生存期（progression-free survival, PFS）^[2-4]^，但因其严重的毒副反应使其疗效进入瓶颈时期。近年来，随着分子生物学的发展，NSCLC中越来越多的基因位点突变被人们所认识和理解，分子靶向治疗的概念随之兴起。其中表皮生长因子受体（epidermal growth factor receptor, EGFR）、间变淋巴瘤激酶（anaplastic lymphoma kinase, ALK）、c-MET等靶向位点更是受到人们极大的关注。目前，EGFR和ALK抑制剂已经被多项Ⅱ期/Ⅲ期临床试验证实对带有*EGFR*敏感性突变和ALK融合基因阳性的晚期NSCLC患者有显著的疗效^[5-7]^，它们先后被食品药品监督管理局（Food and Drug Administration, FDA）批准用于部分NSCLC患者的一线治疗方案中。近期，关于c-MET抑制剂的临床试验报告纷纷显示其可以使NSCLC部分患者生存获益。因此，本文就HGF和c-MET抑制剂治疗NSCLC的研究进展做一综述。

## 2 HGF/c-MET结构和功能

肝细胞生长因子（hepatocyte growth factor, HGF）是由位于7号染色体q21区的基因编码的，包括18个外显子和17个内含子，大小约70 kb。成熟的HGF是由蛋白水解酶作用于其前体产生的一个由96 kD的α链和34 kD的β链经二硫键连接而组成的异二聚体。HGF含6个结构域，分别为氨基末端结构域、4个Kringle结构域及类丝氨酸蛋白酶结构域（SPH）。HGF是一种具有多种功能的生物活性因子，它能作用于多种细胞生长，并参与血管生成及免疫活性的调节。最为重要的是HGF可以促进肿瘤细胞的增殖、转移和侵袭。近年来，研究发现HGF高表达是NSCLC患者使用EGFR-TKI后产生获得性耐药的一个重要原因。

c-MET是由*c-MET*基因编码的一类具有自主磷酸化活性的跨膜受体^[8]^，后被证实HGF是其特异性的配体^[9]^。c-MET的基本结构是由50 kD的α链和145 kD的β链组成的异二聚体，包括3个功能不同的结构域，即胞外区、跨膜区及胞内区。当c-MET与其配体HGF结合后，胞质中酪氨酸残基可发生自身磷酸化，从而激活酪氨酸激酶。随后，细胞质中的多种效应蛋白被快速磷酸化，激活细胞内多种信号通路，如PI3K-Akt、Ras-MAPK及STAT3通路等^[10, 11]^，肿瘤细胞间的相互作用、基质粘附、细胞迁移、侵袭和血管生成。近年来，研究发现HGF/c-MET构成的信号通路在NSCLC的发生和发展中起着重要的作用^[12]^，HGF/c-MET基因过度表达或扩增是EGFR-TKI获得性耐药的一个重要机制^[13, 14]^。

一项对906例NSCLC患者的病理切片应用免疫组织化学和亮视野原位杂交技术检测c-MET表达情况的研究发现，c-MET和p-MET的表达阳性率及*c-MET*基因拷贝数量在低分化腺癌中较高。Sierra等^[15]^研究也发现低分化肺腺癌的患者存在HGF/c-MET高表达，另一项研究也显示高表达的c-MET与NSCLC的乳头状结构相关。这些结果均提示HGF/c-MET在NSCLC发生和发展中起着重要作用。Katayama等^[16]^在建立的吉非替尼耐药细胞系HCC827-GR中检测到*c-MET*基因的扩增，而利用MET-TKI抑制剂（PHA-665752）阻断MET信号通路可以恢复耐药细胞对吉非替尼的敏感性。在临床上，Takeuchi等^[17]^对来自日本的97例NSCLC患者分析发现，HGF过表达占EGFR-TKI获得性耐药的61%，较EGFRT780M二次突变（52%）及*MET*基因扩增（9%）引起的原因更高，而在EGFR-TKI原发耐药的原因中，HGF过表达达到了29%。这些研究均提示HGF/c-MET过表达可能是EGFR-TKI耐药的一个重要原因。

## 3 c-MET靶向抑制剂

目前临床上的c-MET抑制剂可以分为两大类：单克隆抗体和小分子激酶抑制剂。其主要机制均是抑制HGF/c-MET信号通路的传导。单克隆抗体主要针对HGF受体c-MET，一些抗体靶向循环配体HGF。大多数激酶抑制剂靶向多种激酶，只有少数为选择性c-MET激酶抑制剂。

### 3.1 c-MET受体激酶抑制剂

#### 3.1.1 Tivantinib（ARQ197）

Tivantinib（ARQ197）类似于长春新碱，可以阻断细胞周期G_2_期-M期。它不仅可以抑制细胞增殖，还可以诱导c-MET肿瘤细胞的凋亡^[18]^。Tivantinib对未活化的c-MET具有较强的选择性抑制作用，并且可抑制c-MET的自我磷酸化。一项纳入了167例患者的Ⅱ期研究评估了厄洛替尼+tivantinib对比厄洛替尼+安慰剂二线和三线治疗NSCLC的疗效，研究结果显示：两组PFS分别为3.8个月和2.3个月（HR=0.81, 95%CI: 0.57-1.16, *P*=0.24），OS分别为8.4个月和6.8个月（HR=0.88, 95%CI: 0.6-1.3, *P*=0.50）。非鳞癌患者中，PFS分别为4.3个月和2.2个月（HR=0.71, 95%CI: 0.46-1.1, *P*=0.12），OS分别为9.9个月和6.8个月（HR=0.72, 95%CI: 0.6-1.3, *P*=0.18）。两组的主要终点PFS没有达到统计学差异。但是，探索性生存分析显示厄洛替尼+tivantinib组在非鳞癌，*EGFR*野生型和*KRAS*突变患者中PFS和OS有获益的趋势^[19]^。基于这些结果，一项计划纳入988例的Ⅲ期（MARQUEE）随机试验在非鳞癌NSCLC患者中对比了厄洛替尼+tivantinib和厄洛替尼+安慰剂的疗效，初步数据已经在2013年欧洲癌症大会上公布，结果没有达到主要终点OS的延长，但分子亚型分析正在进行中，以确定MET过表达患者能否获益。最常见的不良反应是低级皮疹、腹泻、乏力、恶心、呕吐、呼吸困难、贫血^[20]^。

#### 3.1.2 Cabozantinib

Cabozantinib（XL184/BMS-907351）是一种靶向MET、VEGFR2、AXL、Tie2、KIT、FLT3和RET的多激酶抑制剂^[21]^。该药的临床试验显示其对多种实体瘤具有抗肿瘤作用，包括NSCLC。Cabozantinib于2012年被FDA批准用于治疗进展性转移性甲状腺髓样癌。他的Ⅰ期临床试验显示其与厄洛替尼联用具有较好的安全性及耐受性。随后，一项纳入483例的Ⅱ期临床试验（项目编号：NCT00940225），探究了Cabozantinib对9种实体瘤的治疗效果，结果显示在NSCLC患者中客观缓解率达到40%，其中有13%的患者达到部分缓解（partial response, PR），而存在*EGFR*和*KRAS*突变的NSCLC患者均达到了PR或疾病稳定（stable disease, SD）^[22]^。这提示我们Cabozantinib可能是NSCLC靶向治疗领域中又一种重要的靶向抑制剂。但由于该实验样本量太少，还不足以得出让人信服的结论。

#### 3.1.3 INC280

INC280是一种高效的口服MET抑制剂，它能与酪氨酸激酶抑制剂（tyrosine kinase inhibitors, TKIs）联合治疗*EGFR*突变、MET阳性的NSCLC。一项针对INC280联合吉非替尼的Ⅰb期/Ⅱ期、开放标签、剂量逐步增加的试验（项目编号：NCT01610336），纳入了41例患者，目的是评价INC280联合吉非替尼治疗*EGFR*突变、MET阳性NSCLC的临床疗效。INC280分为100 mg-800 mg *qd*到200 mg-600 mg *bid*的7个剂量队列，联合吉非替尼250 mg *qd*。结果显示有6例出现了局部缓解，其中3例在400 mg *bid*组，5例在入组前接受过EGFR-TKI药物的治疗。所有上述患者均是MET高度激活状态。最常见的药物相关不良反应为恶心（27%）、呕吐、腹泻和皮疹（22%）。最常见的3级/4级不良反应为脂肪酶增加（7%）、淀粉酶增加（5%）。有1例死亡，未排除INC280的原因。以上结果可以看出口服INC280联合吉非替尼的耐受性良好，但推荐剂量尚未确定。初步临床研究结论支持对INC280联合吉非替尼在TKI耐药的MET阳性NSCLC患者中的进一步评估^[23]^。

#### 3.1.4 Crizotinib

目前，Crizotinib更多的被用于ALK阳性的NSCLC患者，由于其对ALK阳性患者显著的治疗效果^[24]^，2011年FDA批准crizotinib用于治疗存在EML4-ALK融合基因的晚期NSCLC患者。但是，crizotinib最早是针对c-MET开发的小分子酪氨酸激酶抑制剂。研究发现crizotinib主要是通过抑制c-MET激酶与ATP的结合及结合的自身磷酸化而发挥作用。一项Ⅰ期临床试验旨在评价crizotinib治疗c-MET扩增晚期NSCLC的疗效和安全性（项目编号：NCT00585195）。研究人员用FISH法将晚期NSCLC患者的c-MET扩增程度分为三类：MET/CEP7比值1.8-2.2为低度扩增，2.2-2.5为中度扩增，≥5为高度扩增。c-MET扩增患者均口服crizotinib 250 mg bid。采用RECIST 1.0标准评价肿瘤客观疗效。结果显示，治疗有效率在低、中、高度扩增患者间分别为0%、17%、67%。常见不良反应包括腹泻（50%）、恶心（31%）、呕吐（31%）、周围性水肿（25%）和视觉障碍（25%），大多数不良事件为1度。该研究初步结果提示我们，c-MET扩增可能代表了一种含新治疗靶点的NSCLC分子亚型，crizotinib对中、高度c-MET扩增的NSCLC患者取得了很好的临床疗效。本研究是单臂试验，未来的临床试验在一线治疗时crizotinib必须与含铂双药方案随机对照，二线时须与单药化疗随机对照。

### 3.2 抗c-MET受体抗体

Onartuzumab（OA-5D5, OAM4558g, MetMAb）是大肠杆菌衍生物，人源化抗MET单克隆抗体。Onartuzumab主要通过抑制HGF/c-MET之间的结合及c-MET二聚化来发挥抗肿瘤的作用。Onartuzumab单药或联合贝伐珠单抗用于晚期实体瘤患者治疗的Ⅰ期临床研究显示出了良好的耐受性。近期公布的一项Ⅲ期随机多中心安慰剂对照的临床试验METLung（OAM4971g），对比了厄洛替尼±onartuzumab应用于Ⅲb期-Ⅳ期NSCLC患者。该研究共纳入499例符合条件的MET免疫组化阳性的患者，主要终点为OS。结果显示，厄洛替尼联合onartuzumab不能改善OS（6.8个月*vs* 9.1个月，HR=1.27，*P*=0.06），PFS（2.7个月*vs* 2.6个月，HR=0.99，*P*=0.92），总体有效率（8.4% *vs* 9.6%，*P*=0.63）。联合治疗组常见不良反应为恶心、气促、背痛、皮疹、周围性水肿、低蛋白血症和痤疮样皮炎，分子亚组的探索性分析还在进行中。尽管onartuzumab在Ⅱ期临床研究中取得了阳性结果（项目编号：NCT00854308），但Ⅲ期临床试验却铩羽而归。当然，该项Ⅲ期试验存在试验设计、方法学、主要研究终点的选取等方面的一些问题，但onartuzumab能否使晚期NSCLC患者的生存获益还需要进一步的研究来证实。

### 3.3 抗HGF单克隆抗体

#### 3.3.1 Ficlatuzumab（AV-299）

Ficlatuzumab（AV-299）是IgG1类人源化抗体，与HGF配体结合具有高亲和力，特异性抑制HGF与c-MET结合。Ficlatuzumab的Ⅰb期临床试验数据表明与EGFR抑制剂厄洛替尼和吉非替尼联合具有良好的耐受性，推荐的Ⅱ期试验剂量为20 mg/kg，每两周1次。一项纳入了未经EGFR-TKI治疗的188例晚期NSCLC患者（*EGFR*突变情况未知）的Ⅱ期临床试验^[25]^，随机分为吉非替尼+Ficlatuzumab和吉非替尼组。结果显示，两者的总体客观缓解率为43% *vs* 40%，中位PFS为5.6月*vs* 4.7月，但是差异无统计学意义。进一步的亚组分析显示吉非替尼+Ficlatuzumab组对c-MET低度表达的患者有效率较高，特别是对EGFR存在激活突变及c-MET低表达两者共存的患者，其PFS获益较大。这提示我们Ficlatuzumab可能延长这类人群对EGFR-TKI耐药的时间。但由于该试验纳入样本量太小，无法得出可靠的结论，仍需要扩大样本量，进行进一步的研究。

#### 3.3.2 Rilotumumab（AMG102）

Rilotumumab（AMG102）是一种完全性IgG2类人源化抗HGF单克隆抗体。主要的作用机制也是抑制HGF与c-MET结合。Ⅰ期临床试验纳入40例难治性晚期实体瘤患者，发现AMG102在高达20 mg/kg的剂量时耐受性也良好。Rilotumumab的Ib期临床试验显示与抗血管生成药物联合使用具有较好的耐受性及安全性^[26]^。几项评估了Rilotumumab联合其他化疗方案和靶向药物在多个肿瘤中的应用的Ⅱ期临床试验也在进行之中（项目编号：NCT 01233687）。

#### 3.3.3 TAK701（HuL 2G7）

TAK701（HuL 2G7）是人源化IgG1单克隆抗体，它主要HGF与c-MET结合以及c-MET受体介导的磷酸化作用。有关研究^[27]^发现TAK701可以和吉非替尼联用，以克服NSCLC患者的对TKI耐药，抑制肿瘤生长。有关TAK701的Ⅰ期研究发现20 mg/kg（两周1次）的剂量具有良好的耐受性，没有剂量限制性毒性，也没有达到最大耐受剂量。最常见的不良事件为咳嗽、腹痛、便秘、疲劳、胃肠道梗阻、胸腔积液、尿路感染和呼吸困难。有关它在NSCLC患者的治疗试验还在探索之中。

## 4 小结

HGF/c-MET信号通路在NSCLC的发生、发展及NSCLC患者对EGFR-TKI的耐药方面都起着重要的作用，其已经成为NSCLC分子靶向治疗的又一热点。有关学者也指出HGF及其c-MET受体有可能成为继*EGFR*、*ALK*之后又一重要的NSCLC驱动基因。HGF/c-MET抑制剂也在临床前的研究中取得良好的抗肿瘤效果。近期公布的一些Ⅱ期/Ⅲ期临床研究的数据显示，HGF/c-MET抑制剂可以使部分c-MET扩增或过表达的NSCLC患者生存获益。但是*c-MET*的突变、扩增和过表达三者之间的关系，究竟何者才是HGF/c-MET信号通路在NSCLC靶向治疗的精确分子靶点，何者才是客观疗效和生存期的最佳预测因子等问题，仍需要更深入的研究来回答。
